# Warming and elevated CO_2_ alter the transcriptomic response of maize (*Zea mays* L.) at the silking stage

**DOI:** 10.1038/s41598-019-54325-5

**Published:** 2019-11-29

**Authors:** Yulan Huang, Rui Fang, Yansheng Li, Xiaobing Liu, Guanghua Wang, Kuide Yin, Jian Jin, Stephen J. Herbert

**Affiliations:** 1 College of Life Science and Technology, Heilongjiang Bayi Agricultural University, Daqing, 163319 China; 2 Key Laboratory of Mollisols Agroecology, Northeast Institute of Geography and Agroecology, Chinese Academy of Sciences, Harbin, 150081 China; 3 Centre for AgriBioscience, La Trobe University, Melbourne Campus, Bundoora, Vic, 3086 Australia; 4 Stockbridge School of Agriculture, University of Massachusetts, Amherst, MA 01003 USA

**Keywords:** Transcriptomics, Abiotic

## Abstract

Exploring the transcriptome of crops in response to warming and elevated CO_2_ (eCO_2_) is important to gaining insights of botanical adaption and feedback to climate change. This study deployed Illumina sequencing technology to characterize transcriptomic profile of maize plants at the silking stage, which were grown under warming (2 °C higher than ambient temperature) and eCO_2_ (550 ppm) conditions. The treatment of ambient temperature and ambient CO_2_ concentration was considered as control (CK). Warming, eCO_2_ and warming plus eCO_2_ resulted in 2732, 1966 and 271 genes expressing differently (DEGs) compared to the CK, respectively. Among the DEGs, 48, 47 and 36 gene ontology (GO) terms were enriched in response to warming, eCO_2_ and warming plus eCO_2_ compared to the CK, respectively. The majority of genes were assigned to the biological process category and the cellular component category. Elevated CO_2_ significantly inhibited gene expressions in terms of photosynthesis and carbohydrate biosynthesis pathways. Warming not only negatively affected expressions of these genes, but also secondary pathways of nitrogen (N) metabolism, including key enzymes of GST30, GST7, GST26, GST15, GLUL and glnA. These results indicated the negative biochemical regulation and physiological functions in maize in response to warming and eCO_2_, highlighting the necessity to improve the genetic adaptability of plant to future climate change.

## Introduction

The increase in atmospheric concentration of CO_2_ is the main driver of global warming. According to the Intergovernmental Panel on Climate Change^1^, atmospheric CO_2_ concentration has exceeded 400 parts per million (ppm) with a consequence of global temperature increase by 0.85 °C since the industrial revolution. The atmospheric CO_2_ concentration is predicted to reach 500~700 ppm by 2050 and 650~1200 ppm by 2100, which is likely to contribute to a global temperature increase of 2 to 4 °C by the end of this century^2^.

Crop production is likely to be influenced by elevated CO_2_ (eCO_2_) and global warming. In general, eCO_2_ stimulates photosynthesis and increases yield in C3 crops while the effect may become less pronounced in C4 crops such as maize^3^ due to greater CO_2_ concentration inside the bundle sheath cells than atmospheric CO_2_ saturating Rubisco^4^. The yield response of maize to eCO_2_ are not consistent among studies with positive^5–7^ or no effect^8^. However, the growth of maize plants may be sensitive to temperature. Using models, Hatfield *et al*. (2016) predicted that every 2 °C increase may result in yield reduction of up to 8% in the Corn Belt region, U.S^9^. In north-west India, Abebe *et al*. (2011) found that the 1.5 °C rise of temperature decreased maize yield by 15%^7^.

A better understanding the mechanisms of eCO_2_ and warming interactive effect on maize yield requires explorations at the genetic level, linking the gene-manipulated physiological functions to plant growth and productivity under climate change. High through-put transcriptome profiling has been widely deployed to identify genes in soybean or maize, which are differentially expressed in response to those environmental factors^10–12^. There were total of 1390 genes in the maize leaf either that were up- or down-regulated during 14 days of exposure to eCO_2_^10^. However, to our knowledge, very few studies have been performed regarding the transcriptome analysis on maize in response to warming plus elevated CO_2_.

With the RNA sequencing technology, we investigated the gene expression profile of maize in response to eCO_2_ and warming at the silking stage which is critical to yield formation. We hypothesized that the expression of genes regulating the photosynthetic pathway might be inhibited by warming, but eCO_2_ might alleviate this negative trend. The results would provide novel data on the biochemical regulation and physiological functions in maize in response to the two environmental factors, which will help to develop better management strategies for crops adapting to future climate changes.

## Materials and Method

### Experimental design and plant growth

A pot experiment was conducted in open-top chambers (OTC) at the Northeast Institute of Geography and Agroecology (45^o^73′N, 126^o^61′E), Chinese Academy of Sciences, Harbin, China. The experimental design was a random block design with two atmospheric CO_2_ concentrations (ambient CO_2_ and eCO_2_) and two temperature levels (ambient temperature and warming). Thus, there were four treatments in total, i.e. ambient CO_2_ and ambient temperature as control (CK), warming only, eCO_2_ only, and warming plus eCO_2_. Each treatment had three replicates. Thus, there were 12 octagonal OTCs. Each OTC had a steel frame body with 2.0 m high and a 0.5 m high canopy, which formed a 45^o^ angle with plane^13^. The OTCs were covered with polyethylene film. The transparency of the film was tested with a portable quantum sensor (MQ-100, Apogee Instruments, Inc. USA) to determine photosynthetically active radiation (PAR), of which the wave length ranges from 409 to 659 nanometers. The photosynthetic photon flux measured inside OTC was more than 95% of the full photo flux outside, indicating that the film cover won’t affect plant photosynthesis in this study. A number of CO_2_-associated studies used similar designs of OTC^14,15^. A digital CO_2_-regulating system (Beijing VK2010, China) was installed to monitor the CO_2_ level inside OTCs and automatically regulated the supply of CO_2_ gas (99.9%) through CO_2_ cylinders to achieve CO_2_ concentrations at 550 ± 30 ppm for eCO_2_ and at 400 ± 30 ppm for ambient CO_2_. An electronic pump was installed at the ground level in each OTC to circulate inner air through a temperature-regulating conditioner system to achieve the OTC-inner temperature as same as ambient temperature or 2 ± 0.5^o^C higher than ambient temperature. The temperature records during plant growth were presented in Fig. S1.

Soil used in this study was collected at Puyang in Henan province (116°52′E, 35°20′N), China. The soil type is Fluvisol^16^. The soil had 18.0 g kg^−1^ of organic C, 1.1 g kg^−1^ of total N, and a pH (1:5 in 0.01 M CaCl_2_) 5.8. The soil was air-dried under a shad and then sieved (≤4 mm). Thirteen kg of the soil was loaded into each pot (30 cm diameter, 35 cm height). Basal nutrients were added into soil at the following rates: 100 mg N kg^−1^ as urea, 50 mg P kg^−1^ and 63 mg K kg^−1^ as KH_2_PO_4_, and 45 mg Ca kg^−1^ as CaCl_2_·2H_2_O. The rates of other nutrients were as follows: 4.2 mg Mg kg^−1^ as MgSO_4_·7H_2_O, 1.7 mg Fe kg^−1^ as Fe-EDTA, 2.2 mg Mn kg^−1^ as MnSO_4_·H_2_O, 2.4 mg Zn kg^−1^ as ZnSO_4_, 2 mg Cu kg^−1^ as CuSO_4_, 0.12 mg B kg^−1^ as H_3_BO_3_, 0.03 mg Co kg^−1^ as CoSO_4_·7H_2_O, and 0.08 mg Mo kg^−1^ as Na_2_MoO_4_·2H_2_O^17^. All pots were allocated into respective OTCs before sowing. Three maize seeds (*Zea mays* L. cv. Xiangyu 998) were sown in each pot and then thinned to 1 plant 10 days after emergence. Soil water content was maintained daily at 80 ± 5% of field capacity by weighing and watering.

### Measurement

At the silking stage (55 days after emergence), approximately 1 g of the top fully extended leaf was sampled from each pot. In particular, the sampling positions were on both sides of the main leaf vein about 20 cm away from leaf tip. The sampling area was similar across plants with about 4 × 7 cm^2^. Leaf samples were immediately frozen in liquid N and stored at −80°C for the RNA extraction. Before RNA extraction, leaf samples were homogeneously ground in liquid N. For chlorophyll measurement, 1 g of fresh leaf on the same position near of each leaf was sampled and ground in alcohol and acetone mixture (1:1). The leaf chlorophyll a and b concentrations in the supernatant of the solution was measured using a spectrophotometer at 663 and 645 nm, respectively^18^. Shoots were also cut at this time at ground level. Then the root system was separated from soil, and washed with tap water to remove soil and sand particles adhering to the roots. Shoot and root samples were oven-dried at 105 °C for 30 min, and then dried at 80 °C for three days. The dry biomass of shoot and root were weighed and recorded. The dried plant samples were ground using a ball mill (Retsol MM2000, Retsch, Haan, Germany) and N concentration was determined by an ELEMENTAR III analyzer (Hanau, Germany).

### RNA extraction, cDNA library construction and sequencing

The leaf RNA was extracted using a RNeasy® Plant Mini kit (Qiagen, Shanghai, China) according to the manufacturer’s instructions. A cDNA library was constructed using a TruSeqt^TM^ RNA Sample Preparation Kit (Illumina, Inc.) and sequenced using an Illumina HiSeq. 2000 platform at Shanghai Majorbio Biopharm Technology Co., Ltd (Shanghai, China). Quality control was performed to eliminate low-quality reads, adaptor polluted reads and ambiguous Ns reads.

### Gene functional annotation and expression analysis

The quality-controlled sequencing data were aligned to the maize genome (NCBI BioProject Accession NO. PRJNA10769). The identification of differentially expressed genes (DEGs) was detected with the Bioconductor package “edgeR” between CK and any of other treatments. The different expression was indicated with *P* value, and the threshold of *P* value was determined by the false discovery rate (FDR)^19^. Genes with *P* ≤ 0.05 and an absolute value of log_2_ fold changes (FC) ≥ 1 were used as the screening criteria^20^. The gene expression among functional groups was analyzed by the Wilcoxon signed-rank test and the influence of CO_2_ and temperature at the transcriptomic level was evaluated. The *P* value ≤ 0.05 was considered as statistical significance.

Differentially expressed genes were further processed with Gene Ontology (GO) function and Kyoto Encyclopedia of Genes and Genomes (KEGG) pathway enrichment analysis. GO and KEGG analyses were performed to identify which DEGs were significantly enriched in GO terms or metabolic pathways. Based on Wallenius non-central hyper-geometric distribution^20^, GO enrichment analysis of the DEGs was conducted using GOseq R packages. GO terms with *P* value < 0.05 were considered significantly enriched among the DEGs. The enriched DEGs in KEGG pathways were analyzed with the KOBAS (v2.0.12) software^21^. A *P* value < 0.05 also was the threshold for DEGs in KEGG pathways. Plant MetGenMAP, a web-based system was used to assign DEGs to metabolic pathways^22^. Sequences of selected unigenes were aligned within the Plant Transcription Factor Database v3.0.

### Gene expression verification by quantitative real time PCR (qRT-PCR)

According to RNA-Seq data, 8 DEGs were randomly selected to verify expressions in sequencing via the qRT-PCR. Selected genes and primers were shown in Table S1. Total RNA (0.3 μg) from each selected gene was treated with gDNA wiper mix and translated into the first strand cDNA, which was stored at −20 °C for the subsequent analysis. All qRT-PCRs were performed in three technical replicates in following steps: pre-denaturation for 5 min at 95 °C and 35 cycles of denaturation for 30 s at 95 °C, annealing for 30 s at 56 °C and extension for 1 min at 72 °C. Outliers were manually discarded. The housekeeping gene GAPDH was used as internal standard to estimate the relative expression. Then the gene expressions were standardized to transcript levels for PtACTIN calculated with the 2^−ΔΔCt^ method^23^.

### Statistical analysis

Data were analysed with Genstat 13 (VSN International, Hemel Hemspstead, UK) including plant biomass, N concentration, chlorophyll concentration, DEGs and KEGG enrichment. One-Way ANOVA tests were used to assess the differences between treatments at significant levels at *P* < 0.05 and *P* < 0.01^24^.

## Results

### Plant biomass, and N and chlorophyll concentrations

Neither warming nor eCO_2_ significantly influenced the shoot dry biomass. A similar result was found in root dry biomass (Table 1). The leaf N concentration was not affected by warming or eCO_2_, but warming plus eCO_2_ resulted in a significant increase in N concentration compared with the CK. Similar trend was found in stem N concentration. Warming did not affect the root N concentration. However, the concentrations of chlorophyll a and chlorophyll a + b were decreased by 20.3% and 18.1% in response to warming compared to the CK. Elevated CO_2_ did not affect chlorophyll concentrations **(**Table 1).

**Table 1:** Effects of warming and elevate CO_2_ (eCO_2_) on plant dry biomass, N concentration and chlorophyll concentrations at the silking stage.

Treatment	Shoot dry biomass (g plant^−1^)	Root dry biomass (g plant^−1^)	Leaf N concentration (mg g^−1^)	Stem N concentration (mg g^−1^)	Root N concentration (mg g^−1^)	Chlorophyll a (mg g^−1^ FW)	Chlorophyll b (mg g^−1^ FW)	Chlorophyll a + b (mg g^−1^ FW)
CK	286 ± 1.11a	53.6 ± 0.96a	15.1 ± 1.01b	9.03 ± 0.52ab	6.35 ± 0.45ab	11.8 ± 0.18a	53.6 ± 0.97a	16.6 ± 0.87a
Warming	293 ± 5.04a	47.3 ± 0.19a	16.1 ± 0.37b	9.41 ± 0.33a	6.84 ± 0.73ab	9.4 ± 1.67b	47.3 ± 0.19a	13.6 ± 2.35b
eCO_2_	317 ± 16.8a	57.0 ± 7.25a	14.1 ± 0.47b	7.68 ± 0.18b	5.63 ± 0.08b	12.3 ± 0.66a	57.0 ± 7.25a	17.2 ± 0.27a
Warming + eCO_2_	301 ± 8.51a	52.2 ± 1.01a	18.7 ± 0.03a	11.5 ± 0.49a	8.16 ± 0.4a	9.4 ± 1.18b	52.2 ± 1.01a	13.1 ± 1.37b

The data are means of three replicates ± standard deviation. Different letters indicate significant difference between treatments (*P* > 0.05). FW represents fresh weight.

### RNA-seq and transcriptomic profile

To comprehensively investigate the transcriptomic and gene expression profiles of maize under eCO_2_ and warming, three cDNA samples extracted from leaves were prepared and sequenced using Illumina NovaSeq. An overview of the RNA-Seq reads of libraries was presented in Table S2. After the low-quality reads were removed, 29 Gb clean reads were obtained with an average of 7.3 Gb reads for each sample, and the proportion of Q30 was greater than 93.33%, which indicates that the sequencing results were highly accurate.

With the Trinity program^25^, the total of 190,928 transcripts were obtained from clean reads. Among them, the length of 99,410 transcripts was more than 1800 bp. The length distribution of transcripts was given in Table S2, suggesting that the sequencing assembly was ideal. The average correlation between the replicates in each treatment reached 0.901. The variation of transcription among replicates was marginally acceptable due to the sampling size of leaf and the influence of other environmental factors such as temperature change during the day.

### Analysis of DEGs and GO annotation

**Figure 1:**
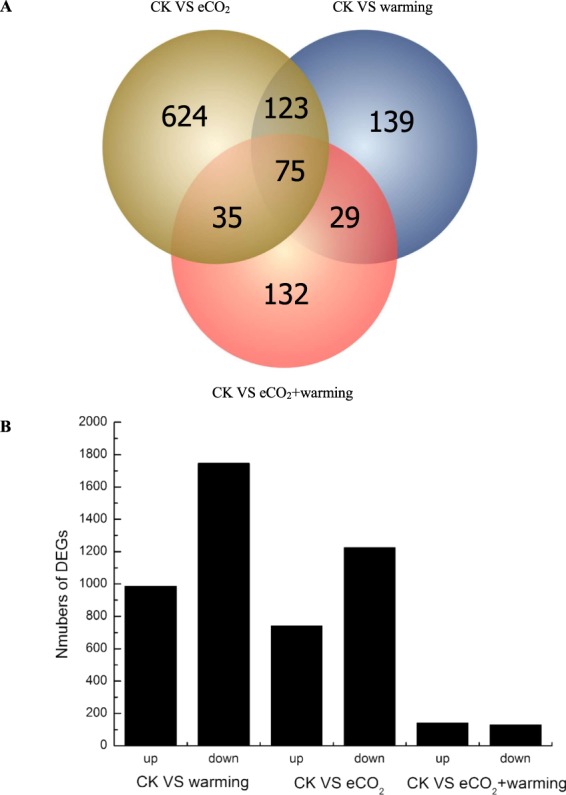
Summary of differentially expressed genes (DEGs) in response to warming, elevated CO_2_ (eCO_2_) and warming + eCO_2_ in a Venn chart (**A**) and up- and down-regulated DEGs in a bar chart (**B**).

Wilcoxon signed-rank test showed that the ratio of DEGs was not more than 1% of the total number of genes in maize. Warming, eCO_2_ and warming plus eCO_2_ caused 2732, 1966 and 271 DEGs compared to the CK, respectively (Fig. 1A). In particular, warming resulted in 986 up-regulated and 1746 down-regulated DEGs, and eCO_2_ led to 741 up-regulated and 1225 down-regulated DEGs. However, only 141 up-regulated and 130 down-regulated DEGs were observed for warming plus eCO_2_ (Fig. 1B). These results indicated that warming and eCO_2_ interactively reduced the expression of numerous genes in maize.

**Figure 2:**
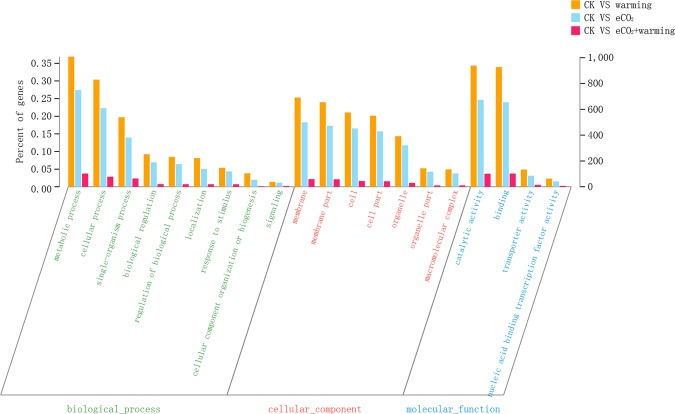
Differentially expressed genes (DEGs) annotated against Gene Ontology (GO) function in response to warming, elevated CO_2_ (eCO_2_) and warming + eCO_2_. The horizontal axis shows the secondary annotation of three primary categories in GO. The left vertical axis indicates the percentage of DEGs to the total annotated genes, and the right vertical axis indicates the number DEGs in this secondary classification of GO.

Among the DEGs, 48, 47 and 37 GO terms were enriched in warming, eCO_2_, eCO_2_ + warming compared with the CK, respectively, which were mainly in biological process, cellular component and the molecular function categories (Fig. 2). In the biological process category, warming and eCO_2_ resulted in 1008 and 749 enriched DEGs respectively, but only 102 for eCO_2_ plus warming. In the cellular component category, there were 691 and 499 of DEGs for warming and eCO_2_, respectively, while only 59 for eCO_2_ plus warming. Regarding molecular function, the numbers of DEGs reached 939 and 673 for warming and eCO_2_, respectively, but only 101 for warming plus eCO_2_.

### KEGG enrichment analysis

**Figure 3:**
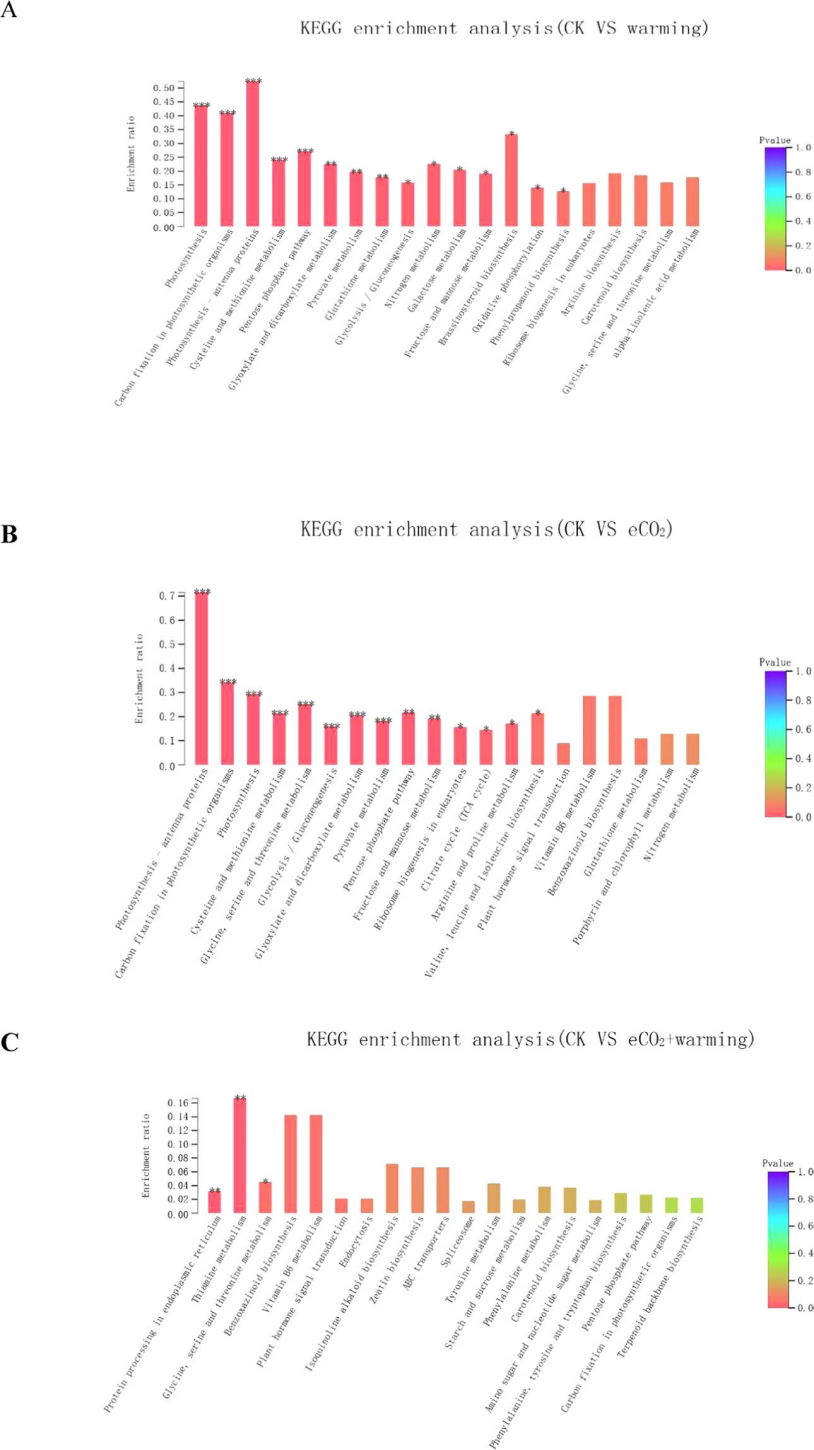
Kyoto Encyclopedia of Genes and Genomes (KEGG) enrichment analysis for the effect of warming (**A**), elevated CO_2_ (eCO_2_) (**B**) and eCO_2_ + warming (**C**). The horizontal axis lists the metabolic pathways, and the vertical axis indicates the proportion of annotated genes in each pathway to the total number of annotated genes. * and ** represent *P* ≤ 0.05 and *P* ≤ 0.01.

To further assess the biological functions of these DEGs, pathway-based analysis was performed using KEGG. Compared to the CK, warming, eCO_2_, and warming plus eCO_2_ significantly enriched 14, 15, and 3 pathways (Fig. 3), respectively. The warming-induced enrichment in metabolic pathways not only included photosynthesis, carbon fixation in photosynthetic organisms, and glyoxylate and dicarboxylate metabolism, but also the secondary metabolisms, such as N metabolism (Fig. 3A). Elevated CO_2_ mainly affected photosynthesis and carbohydrate metabolisms pathways (Fig. 3B). Interestingly, only three metabolism pathways were enriched in response to warming plus eCO_2_. They were plant hormone signal transduction, glycine, serine, threonine, starch and sucrose metabolisms **(**Fig. 3C).

#### Differential expression of photosynthesis-related genes

Compared to the CK, 22 DEGs involved in the photosynthesis pathways, including Photosystem II core complex proteins (psbY), Photosystem I reaction center subunit (psaK) and Photosystem I reaction center subunit N (psaN). Oxygen-evolving enhancer protein 1 (psbO) and Oxygen-evolving enhancer protein 3–1 (psbQ1) were identified as significant down-regulated genes under warming (*P* < 0.05 & |log_2_FC| ≥ 1). Similarly, eCO_2_ significantly altered these DEGs as well. However, eCO_2_ plus warming resulted in only 6 down-regulated genes compared to the CK (Table S3).

#### Differential expression of carbohydrate biosynthesis-related genes

**Figure 4:**
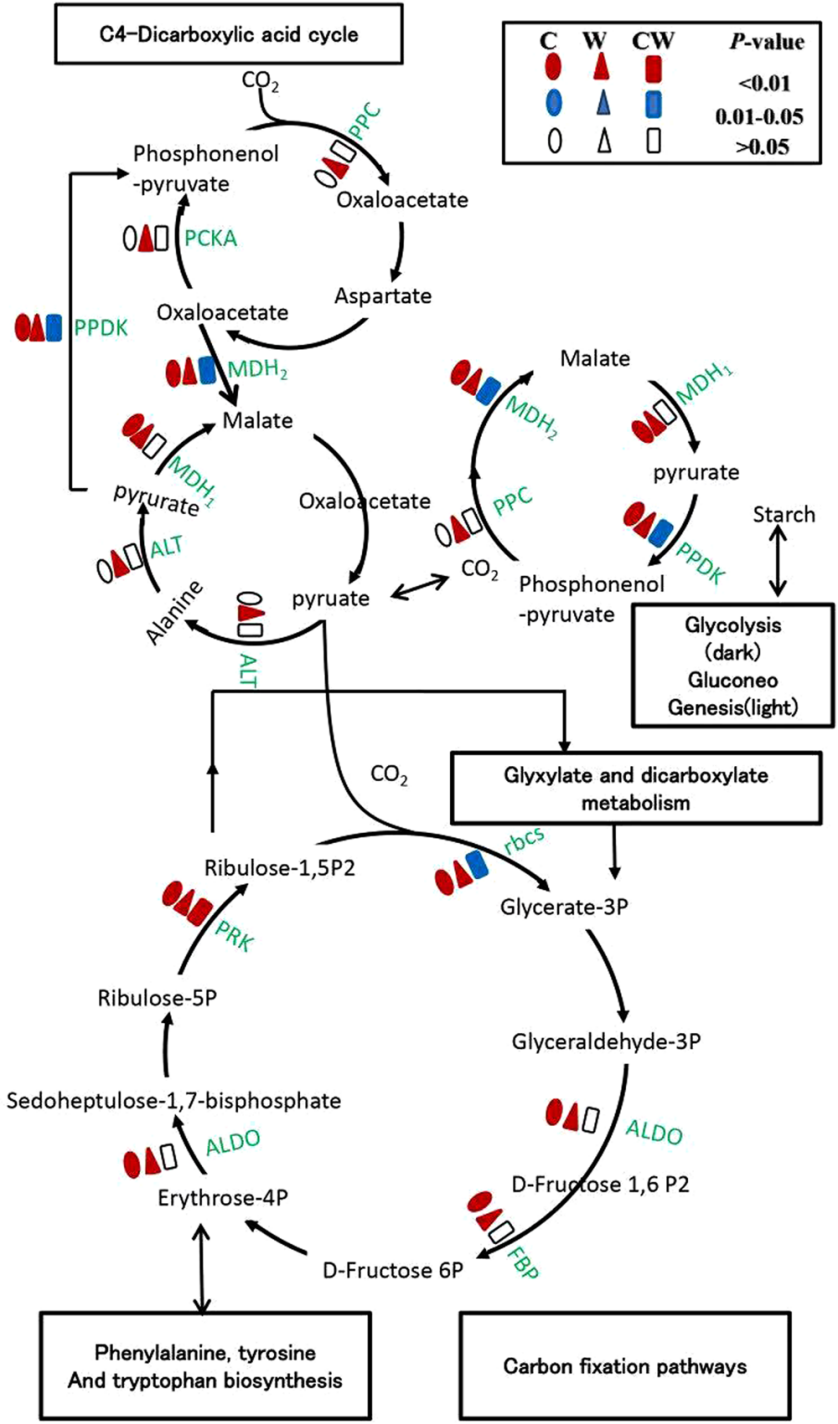
Summary of gene expressions in photosynthesis, sugar, primary and secondary metabolisms in response to warming, elevated CO_2_ (eCO_2_) and warming + eCO_2_. Annotated Genes and enzymes in the metabolic pathway are shown in green letters. C, W and CW represent eCO_2_, warming and eCO_2_ + warming, respectively.

Warming and eCO_2_ had similar effects on carbohydrate biosynthesis-related genes (Fig. 4). Seventeen DEGs in carbohydrate metabolism pathways were significantly down-regulated in response to warming, such as genes responsible for gutathione S-transferase (GST 30), glucose-1-phosphate adenylyltransferase (glgC), fructose-bisphosphate aldolase (ALDO), pyruvate phosphate dikinase (ppdK), gluconokinase (gntK) and glutathione s-transferase (GST). Only two genes were up-regulated, which were associated with pyruvate dehydrogenase (PDHB) and dihydrolipoamide acetyltransferase component of pyruvate dehydrogenase (DLAT). These genes expressed the similar trends in response to eCO_2_ except for the chitinase activity associated genes. Only 7 genes responded to warming plus eCO_2_ (Table S4).

#### Differential expression of primary and secondary metabolism-related genes

In response to warming, 14 DEGs were significantly down-regulated, while 8 were up-regulated in primary and secondary metabolism pathways. Warming resulted in the down-regulated genes including 1-aminocyclopropane-1-carboxylate synthase activity (ACS), terpene synthase activity (TPS11), 12-oxophytodienoate reductase (OPR), cytokinin dehydrogenase (CKX) and lipoxygenase (LOX2S). However, eCO_2_ did not affect these genes. For the up-regulated genes, warming and eCO_2_ had similar effects. There were 10 DEGs, of which 7 were up-regulated expressions and 3 were down-regulated under warming plus eCO_2_ (Table S5).

### qRT-PCR verification for RNA-Seq

To confirm the accuracy of the Illumina RNA-Seq results, 8 of the transcripts related to photosynthesis, sugar and secondary metabolism were selected for qRT-PCR (Table S1). The expression levels of these DEGs with qRT-PCR were compared to those of DEGs with RNA-Seq. A significant correlation (R^2^ = 0.819) was observed between the RNA-Seq and qRT-PCR (Fig. S3). The qPCR results were consistent with their transcript-abundance in RNA-seq, which verified the accuracy of DEGs from RNA-seq analyses in this experiment.

## Discussion

Atmospheric CO_2_ concentration and temperature are expected to be the most important factors impacting the crop production in agriculture^26^. Although maize is the most widely produced crop worldwide, few studies have evaluated the interactive effects of eCO_2_ and warming on transcriptome in relevant to photosynthesis and growth. In this study, we examined the gene expression profiles in transcriptome. Although the RNA expression may not directly alter the synthesis of proteins and relevant metabolisms, the data analysis of gene expression in the present study indicated that temperature and CO_2_ concentration potentially had a significant impact on metabolic pathways such as photosynthesis, C fixation in photosynthetic organisms, and primary and secondary metabolic pathways (Figs. 2–4).

Elevated CO_2_ primarily inhibited gene expression in relation to photosynthesis and sugar metabolic pathways. It was evident that eCO_2_ down regulated the genes coding psbY, psaK, petF, PRK, LHCB1, rbcS, GST30, glgC, ppdK, WAXY, pfkA, PDHB, DLAT, GST 15, ALDO, BX4, MDH1, DLD, GLUL, MDH2, P5CS, POP2 and thrC, which were involved in the Glycolysis, Glyoxylate and dicarboxylate metabolism, fructose and mannose metabolism (Figs. 3 and 4; Tables S3, S4). Although eCO_2_ results in accumulation of photosynthetic carbon assimilation, such as fructose, glucose and sucrose in many C3 species due to eCO_2_-induced stimulation on photosynthesis, and sugar metabolic pathways^5,27–29^, down-regulation of LDH, ALDO, glgC and WAXY genes related to sugar metabolism was found in this study. This down-regulated gene expression in the sugar metabolism may refer to the acclimation responses of maize to eCO_2._ It is probably because a large amount of carbohydrates were accumulated under eCO_2_, inhibiting the relevant sugar metabolisms. The lack of response of biomass to eCO_2_ supported this conclusion (Table 1). In addition, PRK can catalyze the transformation of ribulose-5P into ribulose-1,5P_2,_ which is involved in the glyoxylate and dicarboxylate metabolism. The down regulation of PRK gene would negatively impact the relevant metabolisms.

The eCO_2_-induced inhibition of sugar metabolism was likely to contribute to the down-regulation of photosynthesis pathways. In this study, significant down-regulations in transcripts pertaining to PsaD, PsaF, PsaG, PsaK, PsaO subunit in PSI, and PsbY, Psb27, Psb28, PetE and PetF in PSII were found under eCO_2_. Chen *et al*.^30^ and Ainsworth *et al*.^31^ reported long-term exposure to eCO_2_ can reduce photosynthetic capacity of plants due to accumulation of carbohydrates in the leaf. As C4 crop species have the CO_2_-concentrating mechanism (CCM) in photosynthesis^32^, leaf photosynthesis is closely correlated with the Rubisco. Genes encoding sub-units *rbc*S that are related with Rubisco were inhibited in this experiment. The eCO_2_-induced down-regulations of the expression of genes encoding for sub-units *rbc*S have been observed in other species such as wheat and pea^32–34^. However, the link of photosynthetic capacity with the expression of gene rbcS needs further experimental test under eCO_2_. Moreover, the down regulation of PPDK may affect phosphonend-pyruvate, xaloacetate, and alanine in the C4-dicarboxylic acid cycle (Fig. 3), which is consistent with the findings by Zhang *et al*.^35^.

Warming also negatively affects the expressions of photosynthesis-associated genes **(**Fig. 3, Table S3), contributing to significant down-regulation in transcripts pertaining to PsaD, PsaF, PsaG, PsaK, PsaL, PsaO subunit in PSI and PsbA, PsbO, PsbP, Psb27, Psb28 PetE and PetF in PSII (data not shown). Warming not only affects enzymes in all biochemical reactions associated with photosynthesis, but also the fluidity and integrity of the chloroplast membrane^35^. At the current CO_2_ concentration, Rubisco activity is the main limiting factor for photosynthesis. A study has shown that increasing leaf temperature can affect the expression of Rubisco carboxylase and reduce Rubisco activity, which in turn affects the enzyme activity involved in electron transfer^36^, supporting the results of this study. Although leaf temperature was not measured in this study, a significant relationship between leaf temperature of maize and air temperature (*r* = 0.948, *P* < 0.001) was found in a preliminary study. When the leaf temperature is beyond the optimum temperature range for plant growth, the balance between chlorophyll biosynthesis and catabolism is likely broken, resulting in reduce in leaf chlorophyll content^37,38^. Thus, the increase of leaf temperature due to warming may inhibit the photosynthetic activities^38^. These impaired photosynthesis metabolisms may cause the decrease in chlorophyll concentration of maize in response to warming (Table 1). In addition, warming also fundamentally affects secondary metabolisms such as galactose metabolism, and N metabolism. In particular, the glutamine synthetase (GS) is a key enzyme involved in plant N metabolism, which can catalyze glutamate to synthesize glutamine (Gln). Some Glns are substrates for the production of amino acids in plant^39^. In this study, the expression of GST 30, GST7, GST26, GST 15, GLUL and glnA were significantly down regulated under warming (Tables S4, S5), which may inhibit the N metabolism and the synthesis of amino acids. The slow-down N metabolism under warming may potentially lower N accumulation during the reproductive stage of maize **(**Fig. 3, Table S5). Research showed that warming reduced leaf N concentration of maize, which may be associated with photosynthetic acclimation^26^. Therefore, the change in N metabolism under warming in this study might be another contributor to the reduction of gene expression in relevant to photosynthetic pathways. It is worthy to note that plant performance in pots in this study may be slightly different from the field condition, as the effect of warming on root and soil microbial activity, and subsequent nutrient mineralization and uptake by plants is likely stronger than the field condition. The air warming normally influence the surface soil only in the field. Moreover, the leaf N concentration in this pot experiment (Table 1) was lower than the critical N level in plant^40^. The N deficiency may influence maize metabolisms in response to warming as previous studies showed that low N decreased the production of numerous proteins associated with C and N metabolism and hormonal metabolism at the silking stage of maize^41,42^. Therefore, the plant growth response and relevant gene expression under warming in the pot experiment may differ from field.

In recent years, the interactive effects of warming and eCO_2_ on plant biology have drawn increasing attention^43–45^. However, the number of DEGs in response to warming and eCO_2_ was much less than either the warming or eCO_2_ effect. This may be due to the trade-off effect between warming and eCO_2_ on plant physiological characteristics^46,47^, and consequent non-effect on plants biomass (*p* > 0.05) (Table 1). Ruiz-Vera *et al*.^48^ also reported that there was no significant effect of warming and eCO_2_ on plant biomass of soybean. However, the reason for the trade-off of environmental factors on maize warrants further investigation, which is important to predict how future climate change may affect plant functions.

## Conclusions

This study not only provided transcriptomic datasets of maize at the silking stage under warming and eCO_2_ conditions, but also new biological insights into the expression of genes associated with photosynthesis, carbohydrate biosynthesis and secondary pathways. As a summary diagram showed (Fig. 4), eCO_2_ significantly inhibited gene expressions involved in photosynthesis and carbohydrate biosynthesis pathways. Warming not only negatively affected these genes expression, but also secondary pathways such as N metabolism. The inhibition of these genes in response to warming and eCO_2_ may limit the plant adaptability to future climatic changes.

## Supplementary information

Supplementary section
