# Supplementary material for: Warming and elevated CO2 alter the transcriptomic response of maize (Zea mays L.) at the silking stage
Source: Sci Rep. 2019 Nov 29;9:17948. doi: 10.1038/s41598-019-54325-5 (PMC6884611; doi:10.1038/s41598-019-54325-5)
Supplement: Supplementary file 1 — Supplementary section [file 41598_2019_54325_MOESM1_ESM.docx]

**Warming and elevated CO_2_ alter the transcriptomic response of maize (*Zea mays* L.) at the silking stage**

Yulan Huang, Rui Fang, Yansheng Li, Xiaobing Liu, Guanghua Wang, Kuide Yin, Jian Jin, Stephen J Herbert

**Supplementary tables and figures**

**Figure S1 The average temperature of day time from 6:00 to 18:00 inside OTCs of control (CK) and warming during plant growth**


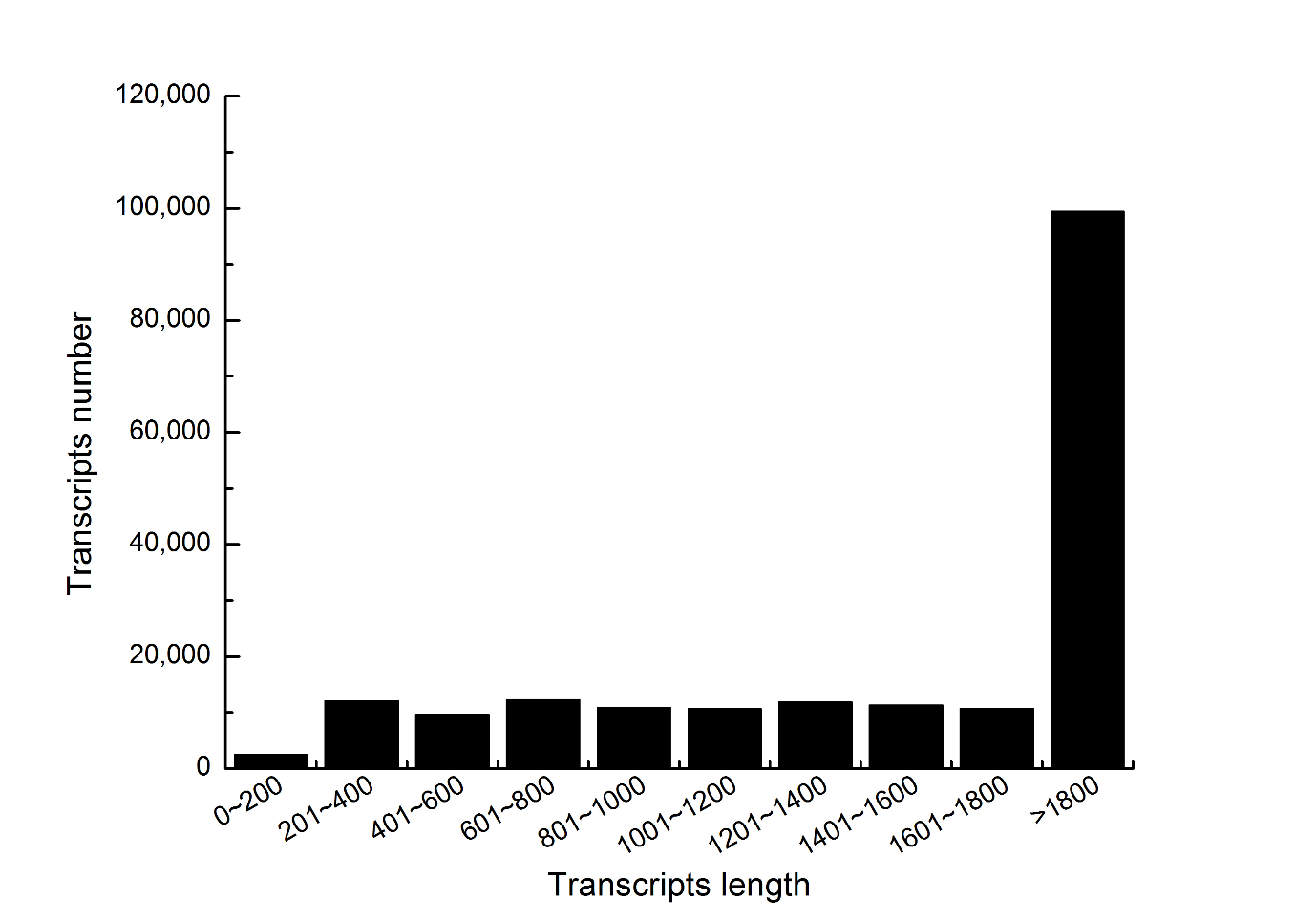


**Figure S2 The length distribution of transcripts**

y=0.879x-0.950

R^2^=0.819

**Figure S3 Quantitative real-time PCR (qRT-PCR) verification for RNA-seq.**

**Table S1 Selected genes and primers for quantitative RT-PCR**

| Gene ID | Gene | Annotation | Fw/Rv | Primer sequence 5’-3’ |
| --- | --- | --- | --- | --- |
| Zm00001d035135 | psbY | Photosystem II core complex proteins | F  R | CGGCCTCCTCCTGCTCATCG  ATGCGGTTGAGCTGGTTGAGC |
| Zm00001d053015 | ALDO | Fructose-bisphosphate aldolase | F  R | TGCCGAGTGCGAGGAGAAGG  GGCTCTGGTTCATGGCGTTCAG |
| Zm00001d045620 | petE | Plastocyanin | F  R | GGCGAGACCTACTCCGTCACC  GATCTTGCCGACCATTCCTGCTC |
| Zm00001d023559 | ALDO | Fructose-bisphosphate aldolase | F  R | CATCCTGCTGAAGCCGTCCATG  TTCTGCGTCGCCTCCACCTC |
| Zm00001d044228 | THI | Thiamine thiazole synthase | F  R | GGCGGTGGAGGACCTGATCG  GACTGCGTGTCGTGGTTCATGG |
| Zm00001d000164 | PRK | Phosphoribulokinase | F  R | CGGACTCCAACACGCTCATCAG  CGCCTTCACCTGCTCGTACATG |
| Zm00001d017711 | PRK | Phosphoribulokinase | F  R | CGGACTCCAACACGCTCATCAG  TCTCCTTCCTGCCGTTCCTGTC |
| Zm00001d010056 | P5CS | glutamate-5-semialdehyde dehydrogenase | F  R | CTTGTGACCTCAGGTGCTGTTGG  GCGAGGAGGACACATCAAGTTGG |

**Table S2 Summary for sequence reads**

| Treatments | Clean reads | Clean bases | GC Content(%) | | | Q30(%) |
| --- | --- | --- | --- | --- | --- | --- |
| CK | 45,391,872~46,179,696 | 6,638,113,375~6,776,751,196 | | 54.63~58.16 | 93.68~94.23 | |
| eCO_2_ | 50,132,260~59,891,866 | 7,364,561,459~8,796,304,389 | | 54.71~55.75 | 94.07~94.27 | |
| Warming | 50,607,362~66,644,084 | 7,432,315,245~9,767597,205 | | 54.40~54.58 | 93.85~94.48 | |
| eCO_2_ + warming | 48,572,206~58,164,018 | 7,114,110,092~8,528,727,928 | | 54.65~61.63 | 93.33~93.82 | |

GC content indicates the percentage of G and C in the total clean bases; Q30 indicates quality score of base is greater than or equal to 30% of the total bases.

**Table S3 Effects of warming, elevated CO_2_ (eCO_2_) and eCO_2_+warming on DEGs associated with photosynthesis**

| Gene ID of maize  genome database | Gene annotation | CK VS warming  Log_2_ FC *P* Value | | CK VS eCO_2_  Log_2_ FC *P* Value | | CK VS eCO_2_+warming  Log_2_ FC *P* Value | |
| --- | --- | --- | --- | --- | --- | --- | --- |
| [Zm00001d035135](http://plants.ensembl.org/Zea_mays/Gene/Summary?g=Zm00001d035135) | photosystem II core complex proteins (psbY) | -5.71 | *P*﹤0.01 | -4.23 | *P*﹤0.01 | -1.26 | *P*﹥0.05 |
| [Zm00001d018797](http://plants.ensembl.org/Zea_mays/Gene/Summary?g=Zm00001d018797) | photosystem I reaction center subunit（psaK） | -3.72 | *P*﹤0.01 | -4.39 | *P*﹤0.01 | -1.67 | *P*﹤0.05 |
| [Zm00001d003767](http://plants.ensembl.org/Zea_mays/Gene/Summary?g=Zm00001d003767) | 16 kDa membrane protein（psaO） | -3.34 | *P*﹤0.01 | -3.62 | *P*﹤0.01 | -1.20 | *P*﹥0.05 |
| [Zm00001d023713](http://plants.ensembl.org/Zea_mays/Gene/Summary?g=Zm00001d023713) | photosystem I reaction center subunit N（psaN） | -2.88 | *P*﹤0.01 | -1.88 | *P*﹤0.05 | -2.66 | *P*﹤0.01 |
| [Zm00001d047532](http://plants.ensembl.org/Zea_mays/Gene/Summary?g=Zm00001d047532) | photosystem II 11 kD protein（psb27） | -3.08 | *P*﹤0.01 | -2.55 | *P*﹤0.01 | -0.568 | *P*﹥0.05 |
| [Zm00001d036535](http://plants.ensembl.org/Zea_mays/Gene/Summary?g=Zm00001d036535) | oxygen-evolving enhancer protein 1（psbO） | -2.46 | *P*﹤0.01 | -1.62 | *P*﹤0.05 | -0.838 | *P*﹥0.05 |
| [Zm00001d021703](http://plants.ensembl.org/Zea_mays/Gene/Summary?g=Zm00001d021703) | oxygen-evolving enhancer protein 3-1（psbQ1） | -2.60 | *P*﹤0.01 | -0.14 | *P*﹤0.05 | -0.423 | *P*﹥0.05 |
| [Zm00001d020877](http://plants.ensembl.org/Zea_mays/Gene/Summary?g=Zm00001d020877) | photosystem I reaction center subunit V（psaG） | -2.43 | *P*﹤0.01 | -2.28 | *P*﹤0.01 | -1.07 | *P*﹥0.05 |
| [Zm00001d005996](http://plants.ensembl.org/Zea_mays/Gene/Summary?g=Zm00001d005996) | photosystem I reaction center subunit V（psaG） | -2.78 | *P*﹤0.01 | -2.63 | *P*﹤0.01 | -2.01 | *P*﹤0.05 |
| [Zm00001d035859](http://plants.ensembl.org/Zea_mays/Gene/Summary?g=Zm00001d035859) | Plastocyanin（petE） | -2.20 | *P*﹤0.01 | -1.37 | *P*﹤0.05 | -0.77 | *P*﹥0.05 |
| [Zm00001d045620](http://plants.ensembl.org/Zea_mays/Gene/Summary?g=Zm00001d045620) | plastocyanin（petE） | -3.75 | *P*﹤0.01 | -2.68 | *P*﹤0.01 | -0.85 | *P*﹥0.05 |
| [Zm00001d049732](http://plants.ensembl.org/Zea_mays/Gene/Summary?g=Zm00001d049732) | ferredoxin-1（petF） | -3.30 | *P*﹤0.01 | -2.32 | *P*﹤0.01 | -1.68 | *P*﹤0.05 |
| [Zm00001d035003](http://plants.ensembl.org/Zea_mays/Gene/Summary?g=Zm00001d035003) | ferredoxin（petF） | -2.18 | *P*﹤0.01 | -2.99 | *P*﹤0.01 | -1.06 | *P*﹥0.05 |
| [Zm00001d000164](http://plants.ensembl.org/Zea_mays/Gene/Summary?g=Zm00001d000164) | phosphoribulokinase（PRK） | -2.45 | *P*﹤0.01 | -2.99 | *P*﹤0.01 | -3.13 | *P*﹤0.01 |
| [Zm00001d017711](http://plants.ensembl.org/Zea_mays/Gene/Summary?g=Zm00001d017711) | phosphoribulokinase（PRK） | -2.87 | *P*﹤0.01 | -3.84 | *P*﹤0.01 | -2.30 | *P*﹤0.05 |
| [Zm00001d026599](http://plants.ensembl.org/Zea_mays/Gene/Summary?g=Zm00001d026599) | chlorophyll a-b binding protein（LHCB6） | -3.33 | *P*﹤0.01 | -2.65 | *P*﹤0.01 | -0.26 | *P*﹥0.05 |
| [Zm00001d044402](http://plants.ensembl.org/Zea_mays/Gene/Summary?g=Zm00001d044402) | chlorophyll a-b binding protein（LHCB1） | -2.33 | *P*﹤0.01 | -3.10 | *P*﹤0.01 | -0.54 | *P*﹥0.05 |
| [Zm00001d044401](http://plants.ensembl.org/Zea_mays/Gene/Summary?g=Zm00001d044401) | chlorophyll a-b binding protein（LHCB1） | -2.33 | *P*﹤0.01 | -3.10 | *P*﹤0.01 | -0.54 | *P*﹥0.05 |
| [Zm00001d033136](http://plants.ensembl.org/Zea_mays/Gene/Summary?g=Zm00001d033136) | chlorophyll a-b binding protein（LHCB2） | -2.17 | *P*﹤0.01 | -2.90 | *P*﹤0.01 | -0.47 | *P*﹥0.05 |
| [Zm00001d033132](http://plants.ensembl.org/Zea_mays/Gene/Summary?g=Zm00001d033132) | chlorophyll a-b binding protein（LHCB2） | -2.17 | *P*﹤0.01 | -2.90 | *P*﹤0.01 | -0.47 | *P*﹥0.05 |
| [Zm00001d046786](http://plants.ensembl.org/Zea_mays/Gene/Summary?g=Zm00001d046786) | chlorophyll a-b binding protein（LHCB2） | -1.96 | *P*﹤0.01 | -3.02 | *P*﹤0.01 | -0.93 | *P*﹥0.05 |
| [Zm00001d052595](http://plants.ensembl.org/Zea_mays/Gene/Summary?g=Zm00001d052595) | ribulose bisphosphate carboxylase small chain（rbcS） | -3.12 | *P*﹤0.01 | -3.48 | *P*﹤0.01 | -2.29 | *P*﹤0.01 |

Log_2_ FC indicates that the difference in quality test expression (fold change) between treatments is converted with log_2_.

**Table S4 Effects of warming, elevated CO_2_ (eCO_2_) and eCO_2_+warming on DEGs associated with carbohydrate biosynthesis metabolism**

| Gene ID of maize  genome database | Gene annotation | CK VS warming  Log_2_ FC *P* Value | | CK VS eCO_2_  Log_2_ FC *P* Value | | CK VS eCO_2_+warming  Log_2_ FC *P* Value | |
| --- | --- | --- | --- | --- | --- | --- | --- |
| [Zm00001d029708](http://plants.ensembl.org/Zea_mays/Gene/Summary?g=Zm00001d029708) | glutathione S-transferase（GST 30 ） | -2.88 | *P*﹤0.01 | -1.68 | *P*﹤0.05 | -0.18 | *P*﹥0.05 |
| [Zm00001d014945](http://plants.ensembl.org/Zea_mays/Gene/Summary?g=Zm00001d014945) | L-lactate dehydrogenase（LDH） | -3.97 | *P*﹤0.01 | -3.04 | *P*﹤0.05 | -1.41 | *P*﹥0.05 |
| [Zm00001d033910](http://plants.ensembl.org/Zea_mays/Gene/Summary?g=Zm00001d033910) | glucose-1-phosphate adenylyltransferase（glgC） | -2.54 | *P*﹤0.01 | -2.46 | *P*﹤0.01 | -2.42 | *P*﹤0.01 |
| [Zm00001d023559](http://plants.ensembl.org/Zea_mays/Gene/Summary?g=Zm00001d023559) | fructose-bisphosphate aldolase（ALDO） | -2.52 | *P*﹤0.01 | -3.77 | *P*﹤0.01 | -1.03 | *P*﹥0.05 |
| [Zm00001d053015](http://plants.ensembl.org/Zea_mays/Gene/Summary?g=Zm00001d053015) | fructose-bisphosphate aldolase（ALDO） | -4.35 | *P*﹤0.01 | -4.02 | *P*﹤0.01 | -1.13 | *P*﹥0.05 |
| [Zm00001d042104](http://plants.ensembl.org/Zea_mays/Gene/Summary?g=Zm00001d042104) | GST7 protein（GST） | -3.55 | *P*﹤0.01 | -2.51 | *P*﹤0.01 | -0.52 | *P*﹥0.05 |
| [Zm00001d043511](http://plants.ensembl.org/Zea_mays/Gene/Summary?g=Zm00001d043511) | hexokinase（HK） | -2.30 | *P*﹤0.01 | -2.03 | *P*﹤0.01 | -1.75 | *P*﹤0.05 |
| [Zm00001d010321](http://plants.ensembl.org/Zea_mays/Gene/Summary?g=Zm00001d010321) | pyruvate phosphate dikinase（ppdK） | -3.56 | *P*﹤0.01 | -4.82 | *P*﹤0.01 | -1.70 | *P*﹤0.05 |
| [Zm00001d050155](http://plants.ensembl.org/Zea_mays/Gene/Summary?g=Zm00001d050155) | gluconokinase（gntK） | -2.97 | *P*﹤0.01 | -2.33 | *P*﹤0.01 | -0.76 | *P*﹥0.05 |
| [Zm00001d049049](http://plants.ensembl.org/Zea_mays/Gene/Summary?g=Zm00001d049049) | pyruvate kinase（PK） | -2.19 | *P*﹤0.01 | -2.38 | *P*﹤0.01 | -2.37 | *P*﹤0.01 |
| [Zm00001d042102](http://plants.ensembl.org/Zea_mays/Gene/Summary?g=Zm00001d042102) | glutathione s-transferase（GST） | -3.45 | *P*﹤0.01 | -3.31 | *P*﹤0.01 | -0.94 | *P*﹥0.05 |
| [Zm00001d019479](http://plants.ensembl.org/Zea_mays/Gene/Summary?g=Zm00001d019479) | starch synthase（WAXY） | -4.19 | *P*﹤0.01 | -3.87 | *P*﹤0.01 | -1.67 | *P*﹤0.05 |
| [Zm00001d024839](http://plants.ensembl.org/Zea_mays/Gene/Summary?g=Zm00001d024839) | glutathione S-transferase 4（GST26） | -2.98 | *P*﹤0.01 | -2.58 | *P*﹤0.01 | -0.21 | *P*﹥0.05 |
| [Zm00001d009936](http://plants.ensembl.org/Zea_mays/Gene/Summary?g=Zm00001d009936) | chitinase activity（CHIB） | -1.99 | *P*﹤0.01 | 0.403 | *P*﹥0.05 | -1.25 | *P*﹥0.05 |
| [Zm00001d008816](http://plants.ensembl.org/Zea_mays/Gene/Summary?g=Zm00001d008816) | ATP-dependent 6-phosphofructokinase（pfkA） | -2.10 | *P*﹤0.01 | -1.82 | *P*﹤0.01 | -0.71 | *P*﹥0.05 |
| [Zm00001d021470](http://plants.ensembl.org/Zea_mays/Gene/Summary?g=Zm00001d021470) | glutathione S-transferase（GST ） | -3.20 | *P*﹤0.01 | -1.40 | *P*﹤0.05 | -1.67 | *P*﹥0.05 |
| [Zm00001d006107](http://plants.ensembl.org/Zea_mays/Gene/Summary?g=Zm00001d006107) | pyruvate dehydrogenase（PDHB） | 2.04 | *P*﹤0.01 | 2.25 | *P*﹤0.01 | 2.09 | *P*﹤0.01 |
| [Zm00001d041839](http://plants.ensembl.org/Zea_mays/Gene/Summary?g=Zm00001d041839) | dihydrolipoamide acetyltransferase component of pyruvate dehydrogenase（DLAT） | 4.29 | *P*﹤0.01 | 3.96 | *P*﹤0.01 | 2.19 | *P*﹤0.05 |
| [Zm00001d010870](http://plants.ensembl.org/Zea_mays/Gene/Summary?g=Zm00001d010870) | glutathione S-transferase（GST 15） | -4.75 | *P*﹤0.01 | -2.88 | *P*﹤0.01 | -1.09 | *P*﹥0.05 |

Log_2_ FC indicates that the difference in quality test expression (fold change) between treatments is converted with log_2_.

**Table S5 Effects of warming, elevated CO_2_ (eCO_2_) and eCO_2_+warming on DEGs associated with primary and secondary metabolisms**

| Gene ID of maize  genome database | Gene annotation | CK VS warming  Log_2_ FC *P* Value | | | CK VS eCO_2_  Log_2_ FC *P* Value | | | CK VS eCO_2_+warming  Log_2_ FC *P* Value | | | | | |
| --- | --- | --- | --- | --- | --- | --- | --- | --- | --- | --- | --- | --- | --- |
| [Zm00001d031449](http://plants.ensembl.org/Zea_mays/Gene/Summary?g=Zm00001d031449) | lipoxygenase（LOX2S） | -2.34 | *P*﹤0.01 | | -0.06 | | *P*﹥0.05 | 1.86 | | | | *P*﹤0.01 | |
| [Zm00001d008862](http://plants.ensembl.org/Zea_mays/Gene/Summary?g=Zm00001d008862) | cytokinin dehydrogenase（CKX） | -3.98 | *P*﹤0.01 | | -1.39 | | *P*﹥0.05 | | 1.54 | | | *P*﹤0.05 | |
| [Zm00001d002326](http://plants.ensembl.org/Zea_mays/Gene/Summary?g=Zm00001d002326) | pyridoxal phosphate（POP2） | -2.14 | *P*﹤0.01 | | -2.17 | | *P*﹤0.01 | | -1.58 | | | *P*﹤0.05 | |
| [Zm00001d044906](http://plants.ensembl.org/Zea_mays/Gene/Summary?g=Zm00001d044906) | 12-oxophytodienoate reductase（OPR） | -2.66 | *P*﹤0.01 | | -1.13 | | *P*﹥0.05 | | -1.67 | | | *P*﹤0.05 | |
| [Zm00001d048703](http://plants.ensembl.org/Zea_mays/Gene/Summary?g=Zm00001d048703) | benzoxazinone synthesis（BX4） | -2.52 | *P*﹤0.01 | | -3.16 | | *P*﹤0.01 | | -0.61 | | | *P*﹥0.05 | |
| [Zm00001d038904](http://plants.ensembl.org/Zea_mays/Gene/Summary?g=Zm00001d038904) | threonine synthase（thrC） | 1.87 | *P*﹤0.01 | | 2.24 | | *P*﹤0.01 | | 2.51 | | | *P*﹤0.01 | |
| [Zm00001d039089](http://plants.ensembl.org/Zea_mays/Gene/Summary?g=Zm00001d039089) | malate dehydrogenase（MDH1） | -2.08 | *P*﹤0.01 | | -1.95 | | *P*﹤0.01 | | -1.07 | | | *P*﹥0.05 | |
| [Zm00001d009779](http://plants.ensembl.org/Zea_mays/Gene/Summary?g=Zm00001d009779) | dihydrolipoyl dehydrogenase（DLD） | 2.14 | *P*﹤0.01 | | 2.37 | | *P*﹤0.01 | | 1.10 | | | *P*﹥0.05 | |
| [Zm00001d024210](http://plants.ensembl.org/Zea_mays/Gene/Summary?g=Zm00001d024210) | terpene synthase activity（TPS11） | -3.91 | *P*﹤0.01 | | -0.90 | | *P*﹥0.05 | | -0.95 | | | *P*﹥0.05 | |
| [Zm00001d003157](http://plants.ensembl.org/Zea_mays/Gene/Summary?g=Zm00001d003157) | S-adenosylmethionine decarboxylase proenzyme（SAMDC） | -2.75 | *P*﹤0.01 | | -2.06 | | *P*﹤0.01 | | -0.42 | | | *P*﹥0.05 | |
| [Zm00001d033747](http://plants.ensembl.org/Zea_mays/Gene/Summary?g=Zm00001d033747) | glutamine synthetase（GLUL） | -4.67 | *P*﹤0.01 | | -2.86 | | *P*﹤0.01 | | 0.20 | | | *P*﹥0.05 | |
| [Zm00001d039487](http://plants.ensembl.org/Zea_mays/Gene/Summary?g=Zm00001d039487) | 1-aminocyclopropane-1-carboxylate synthase activity（ACS） | -5.19 | *P*﹤0.01 | | -1.05 | | *P*﹥0.05 | | -0.54 | | | *P*﹥0.05 | |
| [Zm00001d002258](http://plants.ensembl.org/Zea_mays/Gene/Summary?g=Zm00001d002258) | aminomethyltransferase（AMT） | -2.22 | *P*﹤0.01 | | -2.95 | | *P*﹤0.01 | | -1.43 | | | *P*﹥0.05 | |
| [Zm00001d048050](http://plants.ensembl.org/Zea_mays/Gene/Summary?g=Zm00001d048050) | glutamine synthetase（glnA） | -2.89 | *P*﹤0.01 | | -1.47 | | *P*﹤0.05 | | 0.06 | | | *P*﹥0.05 | |
| [Zm00001d025773](http://plants.ensembl.org/Zea_mays/Gene/Summary?g=Zm00001d025773) | S-adenosylmethionine decarboxylase（AMD1） | -2.62 | *P*﹤0.01 | | -1.85 | | *P*﹤0.01 | | -0.91 | | | *P*﹥0.05 | |
| [Zm00001d044228](http://plants.ensembl.org/Zea_mays/Gene/Summary?g=Zm00001d044228) | thiamine thiazole synthase（THI） | 5.57 | *P*﹤0.01 | | 5.29 | | *P*﹤0.01 | | 4.64 | | | *P*﹤0.01 | |
| [Zm00001d013644](http://plants.ensembl.org/Zea_mays/Gene/Summary?g=Zm00001d013644) | methyltransferase（metE） | 1.98 | *P*﹤0.01 | | 1.28 | | *P*﹤0.05 | | 1.76 | | | *P*﹤0.05 | |
| [Zm00001d022229](http://plants.ensembl.org/Zea_mays/Gene/Summary?g=Zm00001d022229) | L-malate dehydrogenase（MDH2） | -4.50 | *P*﹤0.01 | | -4.87 | | *P*﹤0.01 | | -2.66 | | | *P*﹤0.01 | |
| [Zm00001d010056](http://plants.ensembl.org/Zea_mays/Gene/Summary?g=Zm00001d010056) | glutamate-5-semialdehyde dehydrogenase（P5CS ） | 2.23 | *P*﹤0.01 | | 1.92 | | *P*﹤0.01 | | -0.70 | | | *P*﹥0.05 | |
| [Zm00001d039094](http://plants.ensembl.org/Zea_mays/Gene/Summary?g=Zm00001d039094) | 3-ketoacyl-CoA synthase（KCS） | 4.15 | *P*﹤0.01 | | 4.01 | | *P*﹤0.01 | | 2.00 | | | *P*﹤0.05 | |
| [Zm00001d049187](http://plants.ensembl.org/Zea_mays/Gene/Summary?g=Zm00001d049187) | 6-phosphogluconate dehydrogenase（PGD） | 1.92 | | *P*﹤0.01 | 2.38 | *P*﹤0.01 | | | | 3.12 | *P*﹤0.01 | |  |
| [Zm00001d009779](http://plants.ensembl.org/Zea_mays/Gene/Summary?g=Zm00001d009779) | dihydrolipoyl dehydrogenase（DLD） | 2.14 | | *P*﹤0.01 | 2.37 | *P*﹤0.01 | | | | 1.10 | *P*﹥0.05 | |  |

Log_2_ FC indicates that the difference in quality test expression (fold change) between treatments is converted with log_2_.
